# The Role of Kirschner Wires in Foot and Ankle Surgery: A Comprehensive Review and Practical Appraisal of Applications, Benefits, and Challenges

**DOI:** 10.3390/medicina61101836

**Published:** 2025-10-14

**Authors:** Alberto Arceri, Antonio Mazzotti, Simone Ottavio Zielli, Laura Langone, Federico Sgubbi, Gianmarco Di Paola, Giuseppe D’Antonio, Cesare Faldini

**Affiliations:** 1 1st Orthopaedics and Traumatologic Clinic, IRCCS Istituto Ortopedico Rizzoli, 40136 Bologna, Italy; alberto.arceri@ior.it (A.A.); simoneottavio.zielli@ior.it (S.O.Z.); laura.langone@ior.it (L.L.); federico.sgubbi@ior.it (F.S.); gianmarco.dipaola@ior.it (G.D.P.); giuseppe.dantonio@ior.it (G.D.); cesare.faldini@ior.it (C.F.); 2 Department of Biomedical and Neuromotor Sciences (DIBINEM), Alma Mater Studiorum University of Bologna, 40123 Bologna, Italy

**Keywords:** Kirschner wires, K-wires, deformity, ankle, forefoot

## Abstract

Kirschner wires (K-wires) have remained an integral part of orthopedic surgery for decades, particularly in the management of foot and ankle pathologies. This review examines the role of K-wires by analyzing the applications in fracture fixation and deformity correction, highlighting advantages such as cost-effectiveness and minimal soft-tissue disruption, while acknowledging limitations including lower torsional stability compared with rigid fixation and the risk of pin-tract infection. The purpose was to provide a comprehensive perspective on the clinical applications, advantages, and limitations of K-wires in contemporary surgical practice, with a focus on the most recent evidence from clinical studies.

## 1. Introduction

Kirschner wires (K-wires) have been a cornerstone of orthopedic surgery since their introduction by Martin Kirschner in 1909 [1]. Initially designed for skeletal traction, K-wires revolutionized orthopedic fixation by offering a simple yet robust solution for stabilizing fractures [2]. The application of K-wires in fracture management was first reported by Lambrinudi in 1940, focusing on intramedullary fixation [3]. Over the years, the application of K-wires has expanded beyond trauma cases to include elective surgeries for deformity correction and have evolved into one of the most versatile tools in orthopedic practice [2,4,5,6]. In foot and ankle surgery, K-wires are frequently employed for the treatment of fractures, soft tissue and joint stabilization in deformities, and after osteotomies and arthrodesis procedures [5,7,8,9]. Their ease of use, adaptability, minimally invasive nature, and the ability to be removed have reinforced their clinical relevance. K-wires are typically made of stainless steel or titanium, and are available in various diameters, which enables the user the ability to achieve precision and adaptability [10].

Today, K-wires are often used as temporary fixation tools, aiding in the placement of cannulated screws or serving as preliminary stabilizers before definitive fixation with plates and screws. Their inherent features allow for repeated adjustments to optimize direction and angulation, enabling the surgeon to achieve the desired alignment and correction for the deformity in question [11,12,13,14].

Despite the advent of advanced fixation devices that provide solid, permanent, and reliable stabilization, these innovations come with significantly higher costs, contributing to the financial burden on healthcare systems [10]. In contrast, K-wires remain a cost-effective alternative with proven versatility [15]. While their role has become more marginal in contemporary orthopedic practice, their advantageous characteristics and wide range of applications warrant reconsideration, particularly in appropriately selected patients and surgical procedures.

Anyway, an ongoing debate persists between minimally invasive techniques, often relying on K-wire fixation, and traditional open approaches using rigid internal fixation, particularly regarding their balance of stability, complication risk, and functional recovery. This review examines current clinical applications, advantages, and limitations of K-wires, emphasizing their enduring value and the need to reassess their role in contemporary surgical practice.

## 2. Methods

A targeted search of PubMed/MEDLINE and Scopus was performed through 31 July 2025 to identify clinical and biomechanical studies pertinent to the use of K-wires in foot and ankle surgery. Search terms combined “Kirschner wire” OR “K-wire” OR “K wire” with anatomical and procedural terms (“ankle”, “talar”, “calcaneus”, “midfoot”, “Lisfranc”, “metatarsal”, “phalanx”, “osteotomy”, “arthrodesis”). We included clinical series, comparative observational studies, randomized trials, systematic reviews, and relevant biomechanical studies published in English. Case reports were included only when addressing rare injuries for which higher-level evidence is lacking.

## 3. Clinical Application of Kirschner Wires in Foot and Ankle Surgery

K-wires have a multifaceted role in foot and ankle surgery, reflecting their adaptability and effectiveness across various orthopedic challenges. The unique biomechanics of the foot and ankle region demand versatile solutions for fixation and stabilization, and K-wires meet these requirements by combining ease of use with surgical precision.

Currently, K-wires in foot and ankle surgery are indicated for fracture management, soft-tissue and joint-based stabilization of closed or open reductions, osteotomies, and arthrodesis in various anatomical sites (Table 1).

### 3.1. Fracture Management

#### 3.1.1. Ankle Fractures

Displaced ankle fractures are treated with open reduction and internal fixation (ORIF) [16,17,18]. However, in selective cases, such as severe soft-tissue injuries, fixation using K-wires offers an effective and minimally invasive method for stabilizing fractures. This technique facilitates precise fracture alignment while minimizing surgical exposure, thereby preserving soft tissue integrity and vascular supply, which are crucial for promoting natural healing processes [19,20,21]. For instance, in ankle fracture dislocations, careful soft-tissue management is critical, and external fixation carries risks of recurrent dislocation, infection, and pin-related fractures. To address these limitations, a less invasive percutaneous K-wire fixation was proposed [19,21]. A study specifically investigated the optimal trajectory for calcaneotibial K-wire fixation in the emergent management of unstable ankle fractures [22].

Furthermore, the use of K-wires has been documented in certain types of ankle fractures: a recent retrospective study comparing percutaneous K-wires and cannulated screws for displaced transitional distal tibia fractures found both techniques to provide excellent radiographic, clinical outcomes, and minimal complications, confirming that K-wires represent a safe and efficient alternative to screw fixation. However, K-wires required supplementary stabilization with longer plaster casting compared to the strong stability of the internal fixation [20]. K-wire application is also indicated for certain pediatric fractures, including medial distal tibial Salter-Harris type 3 and 4 epiphyseal injuries. This is to prevent damage to the growth plate [23].

#### 3.1.2. Talar Fractures

Regarding talar fractures, the exclusive use of K-wires for open reduction and internal fixation of major talar fractures is not recommended, as biomechanical studies have demonstrated the superiority of posterior-to-anterior screw placement over anterior-to-posterior fixation or any K-wires combination [24,25].

While not the standard for definitive fixation, K-wires remain a versatile and valuable adjunct in selected scenarios of talar fracture management. In the management of talar neck and body fractures, K-wires are most frequently employed for temporary fixation, allowing surgeons to achieve and maintain reduction intraoperatively before definitive osteosynthesis. Their versatility allows repeated repositioning, which is particularly useful in complex fracture patterns [26]. K-wires may be used adjunctively with threaded screws to enhance rotational stability [25,26,27] and in cases of marked additional ligamentous instability [26]. In selected cases, especially when the fragment size is too small to accommodate a screw or small osteochondral fragments, K-wires may serve as the only feasible fixation method [26]. For instance, a study was conducted that reported the successful treatment of a pediatric comminuted talar fracture using minimal K-wire fixation, with the aim of preventing avascular necrosis. The study found that this treatment method resulted in a favorable functional outcome at two years of follow-up [28].

#### 3.1.3. Calcaneal Fractures

Several studies have documented satisfactory results in terms of patient-reported outcome measures (PROMs), maintenance of fracture reduction at follow-up, and a low complication rate for minimally invasive approaches to the treatment of calcaneal fractures. These approaches involve the reduction and stabilization of fractures through percutaneous K-wire fixation [29,30,31,32,33,34]. The use of the sinus tarsi approach combined with K-wire fixation has been reported as a safe and effective option for the treatment of calcaneal fractures, including in the pediatric population [35,36].

However, current evidence in the literature regarding fixation methods for calcaneal fractures remains inconsistent, making it difficult to establish the clear superiority of one type of hardware over another. In general, no single device provides complete reliability in achieving and maintaining reduction, and fixation strategies should therefore be individualized based on patient characteristics, fracture morphology, and soft-tissue conditions [37].

Most studies agree that K-wire fixation can achieve functional outcomes comparable to those of ORIF, with no significant differences across Sanders fracture types. Moreover, K-wire fixation offers additional advantages, including shorter time to surgery, reduced operative time, lower intraoperative blood loss, shorter hospital stay, faster wound healing, and fewer wound complications [16,29,38,39,40,41,42]. Radiographic outcomes are more debated: while some reports suggest that ORIF provides better joint congruity [43] and superior restoration of parameters such as the angle of Gissane, calcaneal height, and width [16], the majority of studies demonstrate no significant differences between K-wires and other fixation methods in the restoration and maintenance of radiological parameters, including Böhler’s angle, angle of Gissane, calcaneal width, height, length, or varus alignment [29,38,39,41].

In the setting of open calcaneal fractures, K-wires, plate fixation, and cannulated screws appear to be equally effective and well-tolerated [44]. Furthermore, in cases of complex trauma with severe soft-tissue compromise, such as complete hindfoot degloving, temporary stabilization and tissue preservation may be successfully achieved using multiple anchoring K-wires, representing a simple yet valuable technique [45].

#### 3.1.4. Midtarsal Fractures

Fracture-dislocations of the tarsal navicular are rare and complex midfoot injuries, and consequently, most available evidence consists of isolated case reports. Due to the limited number of reported cases and the lack of comparative studies, the optimal surgical strategy remains undefined. While some authors advocate rigid internal fixation with plates and screws, others favor percutaneous pin fixation after open reduction in order to minimize articular damage [46,47,48,49,50]. Microangiographic studies have demonstrated that the central third of the navicular is avascular [51]. This anatomical feature likely accounts for the high risk of nonunion and avascular necrosis associated with navicular fracture-dislocations. Preserving vascularization and preventing avascular necrosis should therefore be prioritized, which supports the use of less aggressive fixation techniques such as K-wires. Notably, one study described the use of two diverging K-wires inserted plantarly, providing fracture stabilization while simultaneously acting as joint blocks, thereby preventing recurrent dislocation and additional articular injury [48].

Due to the cuboid’s robust ligamentous attachments and saddle-shaped articulations, isolated dislocations and fracture-dislocations are exceedingly rare and have been only sporadically reported in the literature. These injuries more commonly occur as part of complex midfoot trauma involving multiple tarsal bones and joints [52,53]. Currently, a consensus regarding the optimal fixation strategy remains elusive, with the debate centering on the relative merits of rigid internal fixation versus percutaneous stabilization. Nonetheless, most authors emphasize the importance of preserving the inherent flexibility of the lateral column of the midfoot, a consideration that supports the use of percutaneous fixation after open reduction, both in cases of fracture and dislocation [52,54,55,56].

Lisfranc injuries are fracture–dislocations of the tarsometatarsal joint complex. A recent multicenter retrospective study conducted by the TRON group compared K-wire fixation with screw fixation, concluding that both techniques provided comparable functional outcomes [57]. Even a review of the mid- and long-term consequences of surgically treated Lisfranc injuries [58] highlighted the study by Marin-Peña [59], which reported the longest available follow-up, with a mean of 180 months in a series of 32 patients predominantly managed with K-wire fixation. In this cohort, the mean AOFAS score at final follow-up was 91, the highest reported for a Lisfranc injury series.

K-wire fixation was also associated with shorter operative times and fewer hardware-related complications, but carried a higher risk of malunion [57]. However, current evidence does not demonstrate the superiority of one fixation technique over another; instead, postoperative outcomes are primarily determined by the achievement of anatomical reduction [60]. Most authors agree that open reduction and internal fixation of the first three metatarsal rays with screws represents a reliable strategy for their management, with supplemental K-wire fixation of the fourth and fifth rays recommended in cases of residual instability, although some surgeons prefer this approach routinely [61]. Other authors employ K-wires indiscriminately across all rays [62].

The role of K-wires has also been emphasized in open Lisfranc injuries, where multiple pin fixation may be advantageous in the presence of comminution and soft-tissue loss. In these cases, the goal is not necessarily anatomical reduction, but rather the maintenance of alignment with multiple wires and the facilitation of early soft-tissue coverage [63]. Moreover, K-wire fixation seems to be used more frequently in younger patients, in whom mid-term outcomes have been reported as more favorable compared to adults [64,65].

#### 3.1.5. Metatarsal and Phalanges Fractures

In the treatment of metatarsal shaft and neck fractures, a recent systematic review reported that K-wire fixation is associated with superior outcomes compared to open reduction and internal fixation, primarily due to a significantly shorter time to fracture healing [66]. Percutaneous K-wire fixation may be performed using antegrade, retrograde, or combined antegrade/retrograde intramedullary techniques. Among these, retrograde percutaneous pinning through the metatarsal head is the most employed method. This typically involves the insertion of a single K-wire along the medullary canal, crossing the fracture site, and, when additional stability is required, extending across the tarsometatarsal joint [66,67,68,69]. Although additional K wires can be used for added strength, this is rarely necessary. Alternative percutaneous methods, such as transverse pinning of the metatarsal heads, have also been described [70,71].

K-wires are particularly useful in cases of multiple metatarsal involvement [2]. In these cases, reduction and stabilization of the most displaced fracture frequently results in anatomical or near-anatomical alignment of the adjacent metatarsals, often obviating the need for further fixation [69].

While toe fractures represent a common foot fracture, especially in the pediatric population, the majority of cases can be managed non-operatively. In cases requiring surgical intervention, K-wires remain an essential option for stabilizing complex injuries, including significantly displaced and intra-articular phalangeal fractures [72,73], Salter–Harris fractures [74,75], and persistent interphalangeal joint fracture-dislocations [76], thereby ensuring stability and reducing the risk of post-traumatic deformity. However, given the small size of the fragments, achieving and maintaining anatomical reduction can be particularly challenging, with an associated risk of revision surgery, refracture, early-onset arthritis necessitating interphalangeal joint fusion, or even avascular necrosis of the fragment [77].

Hallux fractures require surgical intervention more frequently than fractures of the lesser toes, owing to the critical role of the first ray in weight-bearing, balance, and gait mechanics. When closed reduction is selected, stabilization can be achieved using two or more K-wires. This technique is particularly recommended in patients with compromised soft-tissue conditions or significant medical comorbidities [78].

### 3.2. Soft-Tissue and Joint-Based Stabilization Using K-Wires After Closed or Open Reductions

In general, K-wires can be utilized to transfix joints, thereby maintaining bony alignment in cases that would otherwise remain unstable, such as post-reduction for dislocations [79]. As previously mentioned, K-wires serve as essential stabilizing devices following closed or open reduction in isolated traumatic ankle dislocations. In these situations, one or more wires are inserted to secure the ankle in the appropriate anatomical position [19,20,21]. Yang et al. described K-wires employed as an indirect repair for the medial deltoid ligament of the medial tibiotalar joint in combination with ORIF, reporting a significant narrowing of the medial clear space and improved clinical scores [80]. The rationale lies in providing temporary mechanical support to compensate for soft tissue insufficiency while preserving ligament healing potential. Similarly, in subtalar dislocations, particularly when there is a tendency toward redislocation, transfixation of the talonavicular joint with a K-wire is recommended [81]. This approach has also been described in cases of cuboid dislocation [54,55]. Likewise, irreducible metatarsophalangeal joint dislocations of the lesser toes can be managed with reduction followed by temporary transarticular K-wire fixation, especially in the presence of significant joint instability [82]. 

K-wires are integral in managing pediatric foot deformities, where their simplicity and adaptability make them ideal for temporary fixation. Studies have underscored the effectiveness of K-wires in treating conditions like clubfoot, with outcomes demonstrating significant improvements in function and alignment [83,84].

K-wires function both as reduction aids and as temporary stabilizers. In a long-term series of idiopathic clubfeet treated with posterior medial–lateral release, a temporary K-wire was inserted posterolaterally into the malrotated talus to act as a joystick for controlled internal derotation; after reduction in the talonavicular joint, an antegrade K-wire transfixed the talonavicular joint and a second wire was driven from the calcaneal tubercle across the talocalcaneal joint to maintain alignment, with wire removal at six weeks and orthotic management thereafter [85].

In case of congenital convex foot with vertical talus, some authors proposed an open midtarsal release employs a retrograde K-wire inserted into the talar head and retrieved posteriorly through the ankle (without subtalar release), which serves as a lever to elevate and derotate the talus; the wire is then advanced into the navicular to hold the talonavicular reduction, with a second K-wire stabilizing the calcaneocuboid joint [84]. Others suggested Dobbs technique, consisting of a serial manipulation and casting with the so-called reversed Ponseti technique, followed by percutaneous pinning of the talonavicular joint by using K-wire and performing percutaneous tenotomy of the Achilles tendon for the posterior contracture. For incomplete reduction in the talonavicular joint after casting, he recommended an open reduction and K-wire fixation via mini incision [83].

### 3.3. Osteotomies

#### 3.3.1. Calcaneal Osteotomies

Extra-articular joint-preserving calcaneal osteotomies for flatfoot correction can be stabilized using one or more K-wires or alternative fixation methods, such as screws or plates [86].

In pediatric flexible flatfoot or skewfoot, the most commonly performed procedure is a lateral column lengthening, typically achieved through an Evans osteotomy involving an opening wedge osteotomy of the anterior calcaneus with insertion of a bone graft [87]. Fixation strategies for securing the graft have historically ranged from no fixation to percutaneous K-wire stabilization; however, most surgeons advocate for definitive fixation using small-diameter screws, low-profile locking plates, or opening wedge plates [88]. Comparative studies report similar radiographic and functional outcomes between K-wire and plate fixation following Evans osteotomy in symptomatic pediatric flatfoot. Nonetheless, plate fixation has been associated with higher costs and an increased rate of reoperations for hardware-related pain compared to K-wires [89].

In adult flatfoot deformity, the most frequently employed procedure is the medializing calcaneal osteotomy, which generally heals with minimal concern for nonunion. Consequently, some authors support the use of K-wires for temporary stabilization of the osteotomy [86]. Additionally, combined double osteotomy techniques, such as an Evans osteotomy with posterior calcaneal displacement, have been described using temporary K-wire fixation rather than screws, offering advantages including lower cost, technical simplicity, and avoidance of a second procedure for hardware removal [90].

Calcaneal osteotomies are also indicated for correction of hindfoot varus deformities, which can be addressed with a lateral closing wedge osteotomy (Dwyer osteotomy) [91] or a sliding calcaneal osteotomy [92]. Whether performed for clubfoot, cavovarus deformity, or isolated calcaneal varus, these techniques consistently achieve correction of the varus component, with K-wires serving as temporary fixation to maintain alignment during healing [91,92].

#### 3.3.2. Midtarsal Osteotomies

In the surgical management of severe congenital talipes equinovarus after walking age, K-wires play a crucial role in stabilizing the osteotomies and maintaining the corrected alignment of the foot during the postoperative healing phase. A study described a specific technique that consists of a posteromedial release, the Codivilla procedure, combined with cuboid subtraction osteotomy. The osteotomy site was closed and stabilized with a percutaneous K-wire, ensuring proper correction of the forefoot adduction and supination deformities. The wires were left in situ during the initial immobilization period and subsequently removed after 5 weeks at the first cast change. No major complications were reported. Clinical outcomes were good, and no overcorrections were observed [7].

The main midtarsal osteotomy for adult flatfoot is the medial cuneiform opening wedge osteotomy, commonly referred to as the Cotton osteotomy, particularly indicated in cases of forefoot supinatus. Due to the inherent stability provided by the dorsal and plantar ligaments, fixation is often unnecessary; however, when additional stabilization is required, a K-wire may be employed as a temporary fixation device, although some authors reported alternative options, including a dorsal plate or staple [86].

In cases of cavus deformity where tarsectomy (Cole procedure) is deemed necessary, the procedure commonly involves a V-shaped closing wedge osteotomy through the midtarsal region. The apex of the “V” is centered in the navicular bone, and the two limbs of the osteotomy extend laterally toward the cuneiform–cuboid region, just proximal to the first and fifth tarsometatarsal joints. After completing the osteotomy cuts, the distal segment of the foot is translated distally and plantarflexed at the osteotomy site to reduce the excessive arch and correct forefoot pronation. Fixation is typically achieved using K-wires inserted from distal to proximal, maintaining alignment during the healing phase [93].

#### 3.3.3. Metatarsals Osteotomies

The utilization of K-wires in the correction of forefoot deformities, such as hallux valgus and lesser toe deformities, represents an effective, easy, cost-efficient option for maintaining alignment and securing bony fragments during the initial phase of healing.

Various surgical techniques aim to correct the hallux valgus deformity. K-wires are frequently employed as fixation devices to stabilize osteotomies during the bone-healing process [4,8,94,95]. Their straightforward design and ease of use allow surgeons to achieve fixation with minimal equipment, and they are particularly suited to use in minimally invasive surgeries [8,96]. In comparison to other techniques, the use of K-wires in a minimally invasive approach has been demonstrated to yield outcomes that are clinically and radiographically comparable [94,97,98]. Furthermore, some studies suggest that the minimally invasive approach with K-wire provided superior distal metatarsal articular angle (DMAA) correction compared to the open approach; however, available evidence is heterogeneous and largely derived from non-randomized series [94,98].

Their utilization spans proximal and distal osteotomies [4,99].

Although K-wires are often considered biomechanically inferior to other fixation devices, a recent study has demonstrated no significant differences in stability when compared to headless compression screws or absorbable pins [100]. Unlike other fixation methods, K-wires offer tailored and versatile configuration that can be adjusted repeatedly during surgery to meet the specific needs of each case. A finite element study highlighted that K-wire configurations can significantly impact biomechanical outcomes in reducing metatarsal bone stress and enhancing fixation stability, both in procedures such as proximal metatarsal osteotomy (PMO) and distal metatarsal osteotomy (DMO). In particular, the PMO combined with a large cross-angle and shallow insertion fixation (limited to the osteotomy site without penetration into the tarsometatarsal joint) reveals better biomechanical indexes compared to other fixations, while the DMO combined with a single K-wire and shallow insertion fixation reveals better biomechanical indexes compared to other fixations [99].

Metatarsal osteotomies, including shortening procedures for altered metatarsal formulas [8] or Freiberg [101], and one-stage lengthening with bone grafts for brachymetatarsia [9], also benefit from K-wires as they provide support and maintain alignment, enabling effective integration and healing [9,102].

#### 3.3.4. Phalanges Osteotomies

Akin osteotomy can also be performed using minimally invasive techniques with K-wire fixation, achieving comparable correction of the interphalangeal angle to open surgery with screw fixation [103]. The literature also describes configurations using a single K-wire for simultaneous stabilization of multiple osteotomies, such as the concurrent fixation of a DMO and an Akin osteotomy [104].

### 3.4. Arthrodesis

In the context of arthrodesis for conditions like major deformity like severe rigid clubfoot, planus or cavus foot, and post-traumatic sequelae (such as calcaneal fracture), which require subtalar, triple or talo-navicular arthrodesis [5,12,88,105,106], or forefoot deformity (i.e., hallux rigidus, hammer toe) [15,107,108,109], K-wires are widely used to maintain the corrected alignment and stabilize the fusion site during bone healing.

In rigid or relapsed clubfoot, severe cavovarus feet, and pes equinus, K-wires are commonly used to stabilize the correction after wedge resections, Lambrinudi arthrodesis, and triple arthrodesis. After joint preparation, the subtalar, talonavicular and calcaneocuboid joints are transfixed with two or more crossing K-wires to maintain the corrected alignment. This fixation provides sufficient stability during the consolidation phase while allowing relatively simple removal once bone healing has initiated, without the need for permanent implants, which is advantageous in skeletally immature patients [88].

In severe pes planovalgus and joint arthritis (degenerative, inflammatory, and post-traumatic), techniques such as Grice procedure or triple arthrodesis employ K-wires to fix the bone wedge or graft, and stabilize the arthrodesis site [88,105,106].

These procedures using K-wires as a fixation method are particularly suitable for pediatric patients [88], as the fixation is temporary and minimizes implant-related interference with growth. Moreover, wires may help to reduce the risk of wound complications and the requirement for removal of symptomatic hardware, which has been noted to be a problem in some series [105]. Contraindications seem to be low-quality bone and Charcot’s foot. Disadvantages include lower mechanical stability compared to screws or plates and a slightly higher rate of pseudarthrosis at certain joints, particularly the talonavicular joint reported by some series. Nevertheless, the overall fusion rate with K-wire fixation in pediatric foot arthrodesis exceeds 90%, comparable to that achieved with more rigid fixation devices [88]. Anyway, a recent systematic review affirmed that a definitive judgment regarding the best fixation technique is unobtainable from current clinical evidence, due to a lack of high-quality studies [12].

Studies on MTP joint arthrodesis reported a fusion rate using buried k-wire configuration comparable to that achieved with solid crossed screw fixation [6,110,111]. Although it is evident that K-wires are not the most biomechanically robust option for arthrodesis, in patients who require higher load tolerance or more complex corrections, K-wire fixation methods may offer superior outcomes. Furthermore, the ability to adjust K-wire positioning intraoperatively provides surgeons with the flexibility to address unexpected anatomical challenges, which typically occur in these conditions [12,105,107,108]. This highlights the need for tailored treatment strategies based on individual patient needs and surgical goals [6]. Moreover, K-wire’s temporary nature allows for easy removal approximately five to eight weeks following surgery, once the initial fusion phase of callus formation has stabilized the correction. This reduces the risk of long-term implant-related complications and avoids the necessity for the patient to have hardware in situ definitively. This is particularly advantageous in younger patients, who are more likely to require revision procedures in the future.

K-wires are also integral in the surgical correction of lesser toe deformities, providing rigid fixation following proximal interphalangeal (PIP) joint fusion or metatarsophalangeal (MTP) stabilization after tendon transfer or other soft-tissue procedures. They function by maintaining joint immobilization, providing structural support and maintaining the corrected alignment of the toe, ensuring proper healing of surgical adjustments to soft tissues and bony structures. This prevents the recurrence of deformity during recovery [15,107,108,109].

## 4. Discussion

### 4.1. Main Advantages

The enduring utility of K-wires in orthopedic surgery stems from several compelling advantages.

The intraoperative versatility of K-wires remains a significant advantage. Their ability to be easily adjusted during surgery enhances their utility, particularly in complex reconstructions where precise adjustments are necessary [7,72].

In situations of severe soft-tissue compromise or if the patient cannot tolerate longer surgery because of poor general condition, percutaneous K-wire fixation offers a minimally invasive alternative that can stabilize the fracture [26,45]. In general, the main advantages of percutaneous pinning and stabilization are the preservation of bone vascularity, minimal disruption of the soft-tissue envelope, and the avoidance of extensive dissection. However, the limitation is the lack of direct visualization and manipulation of the fracture site [69].

K-wires are easy to insert and remove, making them a practical choice across a wide range of surgical scenarios. Since they leave no internal devices, their removal can be performed in a medical office setting without the need for additional surgical intervention [9].

### 4.2. Economic Impact

K-wires are widely available compared to implants like plates and screws, making them a practical choice in resource-constrained settings [107,109]. Studies reported that K-wires offer an economically favorable option in terms of both the cost of the implant and the equipment required, as well as indirect factors such as the reported reduction in surgical times. This suggests that they represent a more cost-effective approach [15,94,107,108,109].

### 4.3. Complications

The literature reported low complication rates, in terms of tract infections, migration, breakage, recurrence of deformities and malalignment [109,112,113].

The percutaneous insertion of K-wires can expose patients to the risk of pin-track infection, particularly in cases where wires remain in place for extended periods [94,114]. In particular, K-wires allow for reduced exposure of the surgical field compared to ORIF [30,31,38]. Comparative studies have consistently shown that minimally invasive K-wire techniques result in lower infection rates compared to more invasive fixation methods, such as plates and screws, which often necessitate larger incisions and greater soft-tissue dissection [30,31,38]. Percutaneous wire insertion minimizes soft-tissue invasiveness, which is particularly beneficial in fractures and in cases with compromised soft tissue. K-wire insertion minimizes damage to soft tissues and avoid extensive periosteal stripping, maintaining adequate vascular supply to the affected area, both of which are factors associated with a heightened risk of infection. The minimal invasive nature of k-wire not only decreases the likelihood of microbial contamination during surgery but also accelerates recovery [15,94,107,108,109].

Furthermore, protruding wires can interfere with footwear and daily activities and may migrate or break, requiring removal or repositioning. Regular clinical and radiological monitoring is therefore essential to prevent wire migration or loosening [94,107,108,114]. Breakage was exclusive to 1.2 mm K-wires that crossed the MTP joints, while larger K-wires or those not spanning the MTP joint experienced no breakage. Generally, K-wires fail at the proximal point of entry into the metatarsal head. It is typical for fragments to be retained within the metatarsal shaft or head, with no intra-articular fragments that necessitate surgical intervention [109,112]. Long transfixion periods provide superior stabilization, minimizing the risk of deformity recurrence or malalignment. However, prolonged transfixion may compromise joint mobility. A six-week duration is recommended for patients at higher risk of recurrent malalignment, while in cases where joint mobility is a priority, shorter transfixion may be considered. Thus, a customization of treatment is necessary, with patient-specific factors, such as activity level and deformity severity, guiding the choice of transfixion duration [113].

### 4.4. Biomechanical Considerations

Single K-wire fixation may result in instability under torsional forces [99]. From a biomechanical stability perspective, the literature remains inconsistent, most focusing on forefoot applications. While some authors report no significant differences in stability between K-wires and other fixation methods [100], others suggest that K-wires may offer less stability compared to rigid internal fixation devices under high dynamic loads [94]. This underscores the importance of employing a double cross configuration for higher stress anatomical sites and ensuring adequate postoperative immobilization when using K-wires in such scenarios, as the immobilization can affect the early mobilization, ultimately influencing recovery time and the intensity and progression of the rehabilitation program [7,72,115].

## 5. Future Directions

While K-wires continue to demonstrate clinical efficacy in foot and ankle surgery, future research should focus on refining their application through biomechanical optimization, comparing K-wire configurations under physiologic dynamic loading, patient-specific approaches, and improved infection control strategies. Finite element analyses have highlighted the importance of wire configuration and insertion techniques on mechanical performance, suggesting that standardized guidelines for optimal placement could enhance outcomes. Comparative studies, including the newer bioabsorbable materials or headless compression devices, may help delineate specific indications where K-wires retain a clear advantage. Furthermore, a cost-effectiveness analysis integrating implant costs, operative time, and reoperation rates should be performed in order to elucidate the precise cost–benefit profile of K-wire fixation. Finally, prospective randomized multicentric studies with long-term follow-up and standardized PROMs would provide more robust evidence regarding the effectiveness and safety of K-wire fixation.

## 6. Conclusions

K-wires remain a fundamental tool in foot and ankle surgery, balancing simplicity, adaptability, and cost-efficiency. As orthopedic techniques evolve, K-wires are likely to retain their relevance, bridging the gap between traditional methods and cutting-edge technologies. This enduring legacy of K-wires underscores their importance in delivering effective orthopedic care across diverse surgical contexts.

Synthesizing the available literature, K-wires are most appropriate as (1) provisional/joystick fixation during complex reductions, (2) definitive fixation for surgical procedures where implant permanence is undesirable, and (3) temporary stabilizers in soft-tissue–compromised or low-demanding patients with significant comorbidities linked to high risk of developing soft tissue complications. Especially, such scenarios benefit from reduced surgical exposition and faster recovery times, aligning with the needs of these vulnerable patient populations. Evidence from comparative series suggests equivalent short- to mid-term functional outcomes for selected indications, but biomechanical studies indicate inferior torsional stiffness under high dynamic loads for single-wire constructs. Accordingly, K-wires should be used carefully—considering both patient selection and the type of surgical intervention—and with careful postoperative immobilization.

Nevertheless, given the heterogeneity and limited quality of published studies, strong conclusions cannot yet be drawn, and further well-designed comparative studies are required.

## Figures and Tables

**Table 1:** Summary of K-wire Use in Foot and Ankle Surgery.

Anatomical Site	Indication	Role and Advantages	Key Limitations/Complications
Ankle fracture–dislocations	Temporary stabilization after closed/open reduction; soft-tissue compromise	Temporary stabilizer; maintain reduction until soft tissue recovers	Pin-tract infection; need for immobilization; risk of redislocation
Talar neck/body fractures	Adjunct to screws; provisional fixation; not recommended as sole fixation	Adjunctive fixation only; not sole method	Inferior biomechanical stability vs. screws; not suitable for early mobilization
Calcaneal fractures	Temporary stabilization after closed/open reduction; soft-tissue compromise	Comparable outcomes to ORIF; temporary or definitive in selected cases	Plate fixation may reduce malunion risk but higher cost; pin infection
Midtarsal fractures (navicular, cuboid)	Rare fracture–dislocations; temporary fixation after closed/open reduction	Temporary stabilizer; maintain alignment with minimal hardware	Uncertain long-term outcomes
Lisfranc injuries	Adjunct fixation (especially 4th/5th rays)	Adjunct fixation or alternative to screws; lower implant cost	Higher malunion rates vs. screws
Metatarsal fractures/osteotomies	Percutaneous pinning (antegrade/retrograde); temporary fixation after osteotomy	Definitive fixation for single/multiple fractures; simple, percutaneous	No direct visualization of fracture; potential loss of reduction
Toe phalanx fractures (pediatric/adult)	Intra-articular phalangeal fractures, Salter Harris fractures, persistent dislocations	Definitive fixation in complex pediatric fractures; small fragment fixation; prevents deformity	Risk of AVN, arthritis, revision
Flatfoot/cavus osteotomies	Evans/Cotton/MDCO osteotomies; Dwyer/slide osteotomies in cavovarus/flatfoot	Temporary fixation of osteotomy; cost-effective alternative to plates/screws	Requires immobilization; possible loss of correction
Arthrodesis (hindfoot/ankle)	Temporary fixation during fusion positioning; supplemental fixation	Temporary fixation; helps maintain alignment during bone fusion	Not rigid fixation

## Data Availability

Not applicable.

## Abbreviations

The following abbreviations are used in this manuscript:

K-wires	Kirschner wires
ORIF	Open Reduction and Internal Fixation
PIP	Proximal InterPhalangeal
MTP	MetatarsoPhalangeal
PMO	Proximal Metatarsal Osteotomy
DMO	Distal Metatarsal Osteotomy
PROMs	Patient-Reported Outcome Measures
DMAA	Distal Metatarsal Articular Angle

## References

[1] Vécsei V., Hajdu S., Negrin L.L. (2011). Intramedullary Nailing in Fracture Treatment: History, Science and Küntscher’s Revolutionary Influence in Vienna, Austria. Injury.

[2] Patel V., Deshpande S.V., Goel S., Suneja A., Jadawala V.H. (2024). Intramedullary Kirschner Wire Fixation for Metatarsal Fractures: A Comprehensive Review of Treatment Outcomes. Cureus.

[3] Lambrinudi C. (1940). Intra-Medullary Kirschner Wires in the Treatment of Fractures: (Section of Orthopædics). Proc. R. Soc. Med..

[4] Acevedo J.I. (2000). Fixation of Metatarsal Osteotomies in the Treatment of Hallux Valgus. Foot Ankle Clin..

[5] Carlsson Å.S., Önsten I., Besjakov J., Sturesson B. (1995). Isolated Talo-Navicular Arthrodesis Performed for Non-Inflammatory Conditions Blocks Motion in Healthy Adjacent Joints—A Radiostereometric Analysis of 3 Cases. Foot.

[6] Moon J.L., McGlamry M.C. (2011). First Metatarsophalangeal Joint Arthrodesis: Current Fixation Options. Clin. Podiatr. Med. Surg..

[7] Faldini C., Prosperi L., Traina F., Nanni M., Tesfaghiorghi S., Tsegay S., Yosief M., Pungetti C., Sanzarello I. (2016). Surgical Treatment of Neglected Congenital Idiopathic Talipes Equinovarus after Walking Age in Eritrea: An Italo-Eritrean Cooperation. Musculoskelet. Surg..

[8] Giannini S., Faldini C., Nanni M., Di Martino A., Luciani D., Vannini F. (2013). A Minimally Invasive Technique for Surgical Treatment of Hallux Valgus: Simple, Effective, Rapid, Inexpensive (SERI). Int. Orthop..

[9] Giannini S., Faldini C., Pagkrati S., Miscione M.T., Luciani D. (2010). One-Stage Metatarsal Lengthening by Allograft Interposition: A Novel Approach for Congenital Brachymetatarsia. Clin. Orthop. Relat. Res..

[10] Hood C.R., Blacklidge D.K., Hoffman S.M. (2016). Diverging Dual Intramedullary Kirschner Wire Technique for Arthrodesis of the Proximal Interphalangeal Joint in Hammertoe Correction. Foot Ankle Spec..

[11] Tonogai I., Sairyo K. (2021). Temporary Kirschner Wire Fixation of the First Metatarsophalangeal Joint before Osteotomy for Hallux Valgus. Int. J. Surg. Case Rep..

[12] Arumugam V., Ranjit S., Patel S., Welck M. (2023). What Is the Best Fixation Technique for Isolated Talonavicular Arthrodesis?—A Systematic Review. Foot.

[13] Egrise F., Bernard E., Galliot F., Pidhorz L., Mainard D. (2024). Treatment of Two or More Metatarsal Fractures. Orthop. Traumatol. Surg. Res..

[14] Zielli S.O., Mazzotti A., Artioli E., Arceri A., Bonelli S., Ruffilli A., Faldini C. (2023). Retrograde Intramedullary Nail Entry Point for Tibio-Talo-Calcaneal Arthrodesis: A Review of Anatomical Studies. Eur. J. Orthop. Surg. Traumatol..

[15] Albright R.H., Waverly B.J., Klein E., Weil L., Weil L.S., Fleischer A.E. (2018). Percutaneous Kirschner Wire Versus Commercial Implant for Hammertoe Repair: A Cost-Effectiveness Analysis. J. Foot Ankle Surg..

[16] Wu J., Chen Y., Zhu Y., Wu X., Ren P., Cao F. (2023). Clinical Efficacy of Internal Fixation with Locking Compression Plates in the Treatment of Patients with Extremity Fractures and the Effect on the Recovery of Limb Function. Medicine.

[17] Walsh J.P., Hsiao M.S., LeCavalier D., McDermott R., Gupta S., Watson T.S. (2022). Clinical Outcomes in the Surgical Management of Ankle Fractures: A Systematic Review and Meta-Analysis of Fibular Intramedullary Nail Fixation vs. Open Reduction and Internal Fixation in Randomized Controlled Trials. Foot Ankle Surg..

[18] Sutter P.-M., Peltzer J. (2010). Principles of Operative Treatment of Malleolar Fractures Today. Eur. J. Trauma Emerg. Surg..

[19] Xie W., Li H., Zhang C., Cui X., Zhang S., Rui Y., Chen H. (2023). Comparison of Temporary External and Percutaneous K-Wire Fixations for Treatment of Ankle Fracture-Dislocations. BMC Musculoskelet. Disord..

[20] Mishra N., Wang S., Chua Z.K.H., Lam K.Y., Mahadev A. (2021). Comparison of K-Wire versus Screw Fixation after Open Reduction of Transitional (Tillaux and Triplane) Distal Tibia Fractures. J. Pediatr. Orthop. B.

[21] Przkora R., Kayser R., Ertel W., Heyde C.E. (2006). Temporary Vertical Transarticular-Pin Fixation of Unstable Ankle Fractures with Critical Soft Tissue Conditions. Injury.

[22] Schröder M., Stüber V., Walendzik E., O’Loughlin P.F., Zapf A., Krettek C., Gaulke R. (2015). Establishing an Optimal Trajectory for Calcaneotibial K-Wire Fixation in Emergent Treatment of Unstable Ankle Fractures. Technol. Health Care.

[23] Sıvacıoğlu S., Bayram S., Demir T.B., Atasoy I.T., Yoldaş B., Balcı H.I., Aşık M. (2025). Clinical and Radiological Outcomes of Surgically Treated Medial Malleolus Fractures in Skeletally Immature Patients. Ulus Travma Acil Cerrahi Derg..

[24] Swanson T.V., Bray T.J., Holmes G.B. (1992). Fractures of the Talar Neck. A Mechanical Study of Fixation. J. Bone Jt. Surg. Am..

[25] Pajenda G., Vécsei V., Reddy B., Heinz T. (2000). Treatment of Talar Neck Fractures: Clinical Results of 50 Patients. J. Foot Ankle Surg..

[26] Rammelt S., Zwipp H. (2009). Talar Neck and Body Fractures. Injury.

[27] Wohler A.D., Ellington J.K. (2020). Operative Management of a Pediatric Talar Body and Neck Fracture: A Case Report. J. Foot Ankle Surg..

[28] Inal S., Inal C. (2014). A Pediatric Comminuted Talar Fracture Treated by Minimal K-Wire Fixation without Using a Tourniquet. Iowa Orthop. J..

[29] Caravelli S., Gardini G., Pungetti C., Gentile P., Perisano C., Greco T., Rinaldi V.G., Marcheggiani Muccioli G.M., Tigani D., Mosca M. (2023). Intra-Articular Calcaneal Fractures: Comparison between Mini-Invasive Approach and Kirschner Wires vs. Extensive Approach and Dedicated Plate—A Retrospective Evaluation at Long-Term Follow-Up. J. Clin. Med..

[30] Lewis S.R., Pritchard M.W., Solomon J.L., Griffin X.L., Bruce J. (2023). Surgical versus Non-Surgical Interventions for Displaced Intra-Articular Calcaneal Fractures. Cochrane Database Syst. Rev..

[31] Mesregah M.K., Shams A., Gamal O., Zaki E.M. (2020). Clinical and Radiological Outcomes of Minimally Invasive Reduction and Percutaneous K-Wire Fixation for Intra-Articular Calcaneal Fractures. Orthopedics.

[32] Thor J., Socklingam R., Kon C. (2024). Outcomes of Percutaneous Fixation in Intra-Articular Calcaneal Fractures. Cureus.

[33] Morsi I.M., Khalifa A.A., Hussien M.A., Abdellatef A., Refae H. (2020). Evaluation of the Short-Term Results of Closed Reduction and Percutaneous K-Wires Fixation of Displaced Intra-Articular Calcaneal Fractures (DIACF). Foot.

[34] Vittore D., Vicenti G., Caizzi G., Abate A., Moretti B. (2014). Balloon-Assisted Reduction, Pin Fixation and Tricalcium Phosphate Augmentation for Calcanear Fracture. Injury.

[35] Tong L., Li M., Li F., Xu J., Hu T. (2018). A Minimally Invasive (Sinus Tarsi) Approach with Percutaneous K-Wires Fixation for Intra-Articular Calcaneal Fractures in Children. J. Pediatr. Orthop. B.

[36] Wan J., Feng J., Li F., Xu J., Li M.-J., Hu T. (2018). Therapeutic Advantages of Internal Fixation with Kirschner Wire and Bone Grafting via Limited Tarsal Sinus Incision Approach for Displaced Intra-Articular Calcaneal Fractures of Children. Med. Sci. Monit..

[37] Wallin K.J., Cozzetto D., Russell L., Hallare D.A., Lee D.K. (2014). Evidence-Based Rationale for Percutaneous Fixation Technique of Displaced Intra-Articular Calcaneal Fractures: A Systematic Review of Clinical Outcomes. J. Foot. Ankle Surg..

[38] Dai F., Xu Y.F., Yu Z.H., Liu J.T., Zhang Z.G. (2022). Percutaneous Prodding Reduction and K-Wire Fixation Via Sinus Tarsi Approach Versus ORIF for Sanders Type III Calcaneal Fractures: A Prospective Case-Controlled Trial. J. Foot Ankle Surg..

[39] Shams A., Gamal O., Mesregah M.K. (2023). Minimally Invasive Reduction of Intraarticular Calcaneal Fractures With Percutaneous Fixation Using Cannulated Screws Versus Kirschner Wires: A Retrospective Comparative Study. Foot Ankle Spec..

[40] Giannini S., Cadossi M., Mosca M., Tedesco G., Sambri A., Terrando S., Mazzotti A. (2016). Minimally-Invasive Treatment of Calcaneal Fractures: A Review of the Literature and Our Experience. Injury.

[41] Kato M., Takegami Y., Tokutake K., Asami Y., Takahashi Y., Takahashi H., Kumagai H., Imagama S. (2024). Comparison of the Outcomes of Plating, Screw Fixation, and Pinning in Sanders Type II Fractures: A Multicenter (TRON) Retrospective Study. J. Foot Ankle Surg..

[42] Walde T.A., Sauer B., Degreif J., Walde H.-J. (2008). Closed Reduction and Percutaneous Kirschner Wire Fixation for the Treatment of Dislocated Calcaneal Fractures: Surgical Technique, Complications, Clinical and Radiological Results after 2–10 Years. Arch. Orthop. Trauma Surg..

[43] Rickert M.M., McKeithan L.J., Volkmar A.J., Henderson K., Coronado R.A., Mitchell P.M., Gallagher B., Obremskey W.T. (2023). Comparing Calcaneus Fracture Radiographic Outcomes and Complications after Percutaneous Pin versus Screw Fixation. J. Foot Ankle Surg..

[44] Zhao W., Zhang Y. (2019). Comparison and Predictive Factors Analysis for Efficacy and Safety of Kirschner Wire, Anatomical Plate Fixation and Cannulated Screw in Treating Patients with Open Calcaneal Fractures. Medicine.

[45] Herold J., Kamin K., Bota O., Dragu A., Rammelt S. (2023). Complete Avulsion of the Heel Pad with Talar and Calcaneal Fracture: Salvage with Multiple K-Wire Anchorage, Internal Fixation and Free ALT Flap. Arch. Orthop. Trauma Surg..

[46] Mao H., Shi Z., Liu Z., Wang H., Xu D. (2015). Minimally Invasive Technique for Medial Subtalar Dislocation Associated with Navicular and Entire Posterior Talar Process Fracture: A Case Report. Injury.

[47] Singh V.K., Kashyap A., Vargaonkar G., Kumar R. (2015). An Isolated Dorso-Medial Dislocation of Navicular Bone: A Case Report. J. Clin. Orthop. Trauma.

[48] Jimenez I., Rodriguez-Alvarez J.P., Navarro-Navarro R. (2017). Dorsal Fracture-Dislocation of the Tarsal NavicularCase Report and Review of a Rare Injury. J. Am. Podiatr. Med. Assoc..

[49] Lüthje P., Nurmi I. (2002). Fracture-Dislocation of the Tarsal Navicular in a Soccer Player. Scand. J. Med. Sci. Sports.

[50] Hamdi K., Hazem B.G., Yadh Z., Faouzi A. (2015). Isolated Dorsal Dislocation of the Tarsal Naviculum. Indian J. Orthop..

[51] Kennedy J.G., Maher M.M., Stephens M.M. (1999). Fracture Dislocation of the Tarsal Navicular Bone: A Case Report and Proposed Mechanism of Injury. Foot Ankle Surg..

[52] Kaiser P.B., Briceno J., Kwon J.Y. (2019). Complete Cuboid Dislocation with Associated Lisfranc Injury: A Case Report and Review of the Literature. J. Foot Ankle Surg..

[53] Miersch D., Wild M., Jungbluth P., Betsch M., Windolf J., Hakimi M. (2011). A Transcuneiform Fracture-Dislocation of the Midfoot. Foot.

[54] Mazzotti A., Bonelli S., Zielli S., Arceri A., Viglione V., Faldini C. (2021). Traumatic Cuboid Dislocation. The Potential Role of Plantar Ligaments Integrity in Facilitating Reduction: A Case Report. JBJS Case Connect..

[55] de Oliveira Lima A., de Albuquerque Filho A.B., Ambrosio G.H.C., Coelho L.V., Massuda V.Y., Lara P.H.S. (2024). Isolated Cuboid Dislocation: Case Report. J. Surg. Case Rep..

[56] Sassine T.J., Terra B.B., Giordano V., Ejnisman B. (2021). Cuboid Nutcracker Fracture in a 9-Year-Old Child. BMJ Case Rep..

[57] Adachi A., Takegami Y., Nakashima H., Mishima K., Kobayashi K., Imagama S. (2025). Comparative Clinical Outcomes of K-Wire Fixation versus Screw Fixation in Lisfranc Joint Injuries: A Multicenter (TRON Group) Retrospective Study. J. Foot Ankle Surg..

[58] Fontanella L., Schwab J.M., Chidda A., Tannast M., Seidel A. (2024). The Mid- and Long-Term Consequences After Surgically Treated Lisfranc Injuries: A Case Series and Review of the Literature. Cureus.

[59] Marín-Peña O.R., Viloria Recio F., Sanz Gómez T., Larrainzar Garijo R. (2012). Fourteen Years Follow up after Lisfranc Fracture-Dislocation: Functional and Radiological Results. Injury.

[60] Mascio A., Greco T., Maccauro G., Perisano C. (2022). Lisfranc Complex Injuries Management and Treatment: Current Knowledge. Int. J Physiol. Pathophysiol. Pharmacol..

[61] Stavlas P., Roberts C.S., Xypnitos F.N., Giannoudis P.V. (2010). The Role of Reduction and Internal Fixation of Lisfranc Fracture-Dislocations: A Systematic Review of the Literature. Int. Orthop..

[62] Mosca M., Fuiano M., Censoni D., Marcheggiani Muccioli G.M., Roberti di Sarsina T., Grassi A., Caravelli S., Zaffagnini S. (2021). A Mid-Term Follow-up Retrospective Evaluation of Tarsometatarsal Joint Fracture-Dislocations Treated by Closed Reduction and Percutaneous K-Wires Fixation. Injury.

[63] Nithyananth M., Boopalan P.R.J.V.C., Titus V.T.K., Sundararaj G.D., Lee V.N. (2011). Long-Term Outcome of High-Energy Open Lisfranc Injuries: A Retrospective Study. J. Trauma Inj. Infect. Crit. Care.

[64] Sensoz E., Yilmaz H., Onay T. (2025). Mid-Term Radiologic and Clinical Results of Pediatric-Adolescent Lisfranc Injuries. J. Pediatr. Orthop..

[65] Cheow X., Lam K.Y. (2018). Midterm Functional Outcomes in Operatively Treated Adolescent Lisfranc Injuries. J. Pediatr. Orthop. B.

[66] Stavrakakis I.M., Tourvas E.A., Magarakis G.E., Sperelakis I.V., Kristan A., Tosounidis T.H. (2021). Operative Treatment of Acute Shaft and Neck Lesser Metatarsals Fractures: A Systematic Review of the Literature. Eur. J. Orthop. Surg. Traumatol..

[67] Zarei M., Bagheri N., Nili A., Vafaei A., Ghadimi E. (2020). Closed Antegrade/Retrograde Intramedullary Fixation of Central Metatarsal Fractures: Surgical Technique and Clinical Outcomes. Injury.

[68] Samaila E.M., Ditta A., Negri S., Leigheb M., Colò G., Magnan B. (2020). Central Metatarsal Fractures: A Review and Current Concepts. Acta Biomed..

[69] Buddecke D.E., Polk M.A., Barp E.A. (2010). Metatarsal Fractures. Clin. Podiatr. Med. Surg..

[70] Goel N.K., Khurana A., Narula V., Goyal A. (2021). Closed Transverse Pinning for Reduction and Fixation of Metatarsal Neck Fractures: Surgical Technique. Indian J. Orthop..

[71] Moharrami A., Mirghaderi S.P., Hoseini Zare N., Tabatabaei Irani S.P., Moazen-Jamshidi M.M., Kalantar S.H. (2022). Transverse Pinning of Concomitant First and Second Metatarsal Fractures Using 1.5mm K-Wires; Case Report and Technical Note. Ann. Med. Surg..

[72] Kim H.T., Cho Y.J., Kim J.H. (2024). Delayed Correction of Intra-Articular Lateral Head Fracture of the Proximal Phalanx of the Great Toe in Children. Clin. Orthop. Surg..

[73] Robertson G.A.J., Sinha A., Hodkinson T., Koç T. (2023). Return to Sport Following Toe Phalanx Fractures: A Systematic Review. World J. Orthop..

[74] Harris T.A., Krumrey J., Sharp J. (2021). Development of an Effective Treatment Algorithm for the Stubbed Great Toe. Cureus.

[75] Buch B.D., Myerson M.S. (1995). Salter-Harris Type IV Epiphyseal Fracture of the Proximal Phalanx of the Great Toe: A Case Report. Foot Ankle Int..

[76] Veen M., Schipper I.B. (2013). Irreducible Fracture of the Proximal Interphalangeal Joint of the Fifth Toe. J. Emerg. Med..

[77] Kramer D.E., Mahan S.T., Hresko M.T. (2014). Displaced Intra-Articular Fractures of the Great Toe in Children: Intervene with Caution!. J. Pediatr. Orthop..

[78] Godoy-Santos A.L., Giordano V., Cesar C.D., Sposeto R.B., Bitar R.C., Wajnsztejn A., Sakaki M.H., Fernandes T.D. (2020). Hallux Proximal Phalanx Fracture in Adults: An Overlooked Diagnosis. Acta Ortop. Bras..

[79] Taylor R.G. (1962). Immobilization of Unstable Fracture Dislocations by the Use of Kirschner Wires. Proc. R. Soc. Med..

[80] Yang T., Zhu F., Wang H., Wu B., Jia D., Meng C., Zhao Y. (2024). Kirschner Wire Internal Fixation of the Medial Tibiotalar Joint for Indirect Repair of Deltoid Ligament Injury: A Retrospective Comparative Study. Orthop. Surg..

[81] Wagner R., Blattert T.R., Weckbach A. (2004). Talar Dislocations. Injury.

[82] Brunet J.A., Tubin S. (1997). Traumatic Dislocations of the Lesser Toes. Foot Ankle Int..

[83] Grzegorzewski A., Lipiński Ł., Pruszczyński B., Grzegorzewski P., Buchcic P. (2023). Results of Treatment of Congenital Vertical Talus by the Dobbs Method. J. Orthop. Surg. Res..

[84] Ramanoudjame M., Loriaut P., Seringe R., Glorion C., Wicart P. (2014). The Surgical Treatment of Children with Congenital Convex Foot (Vertical Talus): Evaluation of Midtarsal Surgical Release and Open Reduction. Bone Jt. J..

[85] Hsu L.P., Dias L.S., Swaroop V.T. (2013). Long-Term Retrospective Study of Patients with Idiopathic Clubfoot Treated with Posterior Medial-Lateral Release. J. Bone Jt. Surg. Am..

[86] Peterson K.S., Overley B.D., Beideman T.C. (2015). Osteotomies for the Flexible Adult Acquired Flatfoot Disorder. Clin. Podiatr. Med. Surg..

[87] Mazzotti A., Sgubbi F., Arceri A., Di Paola G., Artioli E., Zielli S.O., Marcucci L., Guindani N., Faldini C., De Pellegrin M. (2025). Skewfoot Deformity: State of the Art. Children.

[88] Kreher J., Putz C., Fackler S., Müller S., Horsch A., Geisbüsch A. (2023). K-Wire Osteosynthesis for Arthrodesis of the Paediatric Foot Is a Good and Valid Procedure. J. Clin. Med..

[89] Tippabhatla A., Torres-Izquierdo B., William M., Pereira D., Meyer Z., Hosseinzadeh P. (2023). Is There a Benefit to Rigid Fixation in Calcaneal Lengthening Osteotomy in Painful Pediatric Idiopathic Flatfoot Deformity? Comparing Results of Kirschner Wire Versus Plate Fixation. J. Pediatr. Orthop..

[90] Basioni Y., El-Ganainy A.-R., El-Hawary A. (2011). Double Calcaneal Osteotomy and Percutaneous Tenoplasty for Adequate Arch Restoration in Adult Flexible Flat Foot. Int. Orthop..

[91] Robinson D.S., Clark B., Prigoff M.M. (1988). Dwyer Osteotomy for Treatment of Calcaneal Varus. J. Foot Surg..

[92] Chen Z.-Y., Wu Z.-Y., An Y.-H., Dong L.-F., He J., Chen R. (2019). Soft Tissue Release Combined with Joint-Sparing Osteotomy for Treatment of Cavovarus Foot Deformity in Older Children: Analysis of 21 Cases. World J. Clin. Cases.

[93] Chatterjee P., Sahu M.K. (2009). A Prospective Study of Japas’ Osteotomy in Paralytic Pes Cavus Deformity in Adolescent Feet. Indian J. Orthop..

[94] Kim J., Oh M., Kyeong T.H., Choi M.N., Lee S.Y. (2024). Radiographic Comparison of Open and Minimally Invasive Distal Chevron Metatarsal Osteotomy in Patients with Hallux Valgus. J. Foot Ankle Surg..

[95] Mn B., U B., A T., R D. (2017). A Prospective Study of Distal Metatarsal Chevron Osteotomies with K-Wire Fixations to Treat Hallux Valgus Deformities. Cureus.

[96] Mazzotti A., Zielli S.O., Abdi P., Artioli E., Arceri A., Vannini F., Faldini C. (2023). Severe Hallux Valgus Can Be Treated Using a Distal Metatarsal Osteotomy: Results of 144 Cases Treated with the SERI Technique. Foot Ankle Surg..

[97] Bİlgİn E., KeÇecİ T., Turgut A., Adiyeke L., Kİlİnc B.E. (2020). Comparison of Clinical and Radiological Results of Two Fixation Materials after Distal Chevron Osteotomy for Hallux Valgus?—Two Kirschner Wires versus Single Screw Fixation. Acta Chir. Orthop. Traumatol. Cech..

[98] Giannini S., Cavallo M., Faldini C., Luciani D., Vannini F. (2013). The SERI Distal Metatarsal Osteotomy and Scarf Osteotomy Provide Similar Correction of Hallux Valgus. Clin. Orthop. Relat. Res..

[99] Shih K.-S., Hsu C.-C., Huang G.-T. (2023). Biomechanical Investigation of Hallux Valgus Deformity Treated with Different Osteotomy Methods and Kirschner Wire Fixation Strategies Using the Finite Element Method. Bioengineering.

[100] Kuru T., Mutlu I., Bilge A., Nusran G., Kaymaz B., Yilmaz O., Kizilay H., Ceviz E., Yaradilmis Y.U., Erken H.Y. (2022). Biomechanical Comparison of Headless Compression Screws, Kirschner Wires and Bioabsorbable Pins in Distal Oblique Metatarsal Osteotomy for Correction of Hallux Valgus. J. Am. Podiatr. Med. Assoc..

[101] Yoshimura I., Takao M., Wagner E., Stufkens S., Dahmen J., Kerkhoffs G.M.M.J., Glazebrook M. (2024). Evidence-Based Treatment Algorithm for Freiberg Disease. Cartilage.

[102] Arceri A., Mazzotti A., Zielli S.O., Artioli E., Viroli G., Traversari M., Ruffilli A., Faldini C. (2024). What’s the Evidence on Surgical Treatment for Congenital Brachymetatarsia: A Systematic Review and Meta-Analysis. J. Orthop..

[103] Schilde S., Delank K.-S., Arbab D., Gutteck N. (2021). Minimally Invasive vs. Open Akin Osteotomy. Foot Ankle Int..

[104] Mazzotti A., Zielli S.O., Giacomo C., Artioli E., Arceri A., Abdi P., Langone L., Faldini C. (2024). Combined Distal Metatarsal and Akin Osteotomies for Concomitant Metatarsophalangeal and Interphalangeal Hallux Valgus: Clinical and Radiological Outcomes. J. Foot Ankle Surg..

[105] Ohly N.E., Cowie J.G., Breusch S.J. (2016). Triple Arthrodesis of the Foot with Allograft through a Lateral Incision in Planovalgus Deformity. Foot Ankle Surg..

[106] Napiontek M., Pietrzak K. (2011). Triple Arthrodesis of the Foot after Calcaneal Fractures. Twelve Patients Treated Using K Wires Stabilization. Foot Ankle Surg.

[107] Shirzad K., Kiesau C.D., DeOrio J.K., Parekh S.G. (2011). Lesser Toe Deformities. J. Am. Acad. Orthop. Surg..

[108] Trnka H.-J., Trnka P. (2024). Classical Surgical Alternatives for the Treatment of Lesser Toe Deformities. Foot Ankle Clin..

[109] Kramer W.C., Parman M., Marks R.M. (2015). Hammertoe Correction with K-Wire Fixation. Foot Ankle Int..

[110] Storts E.C., Camasta C.A. (2016). Immediate Weightbearing of First Metatarsophalangeal Joint Fusion Comparing Buried Crossed Kirschner Wires Versus Crossing Screws: Does Incorporating the Sesamoids into the Fusion Contribute to Higher Incidence of Bony Union?. J. Foot Ankle Surg..

[111] Karlock L.G., Berry L., Craft S.T., Petrozzi R., Grahn A.G., Casteel M.L. (2017). First Metatarsophalangeal Joint Fusion with Use of Crossed Kirschner Wires and Intramedullary Steinmann Pin. J. Foot Ankle Surg..

[112] Zingas C., Katcherian D.A., Wu K.K. (1995). Kirschner Wire Breakage after Surgery of the Lesser Toes. Foot Ankle Int..

[113] Klammer G., Baumann G., Moor B.K., Farshad M., Espinosa N. (2012). Early Complications and Recurrence Rates after Kirschner Wire Transfixion in Lesser Toe Surgery: A Prospective Randomized Study. Foot Ankle Int..

[114] Kim J.-S., Ko M.-S., Ri G.-S., Pak S.-G., Kim H.-M., Kim J.-H. (2019). Bioabsorbable Pins Versus Stainless Steel Kirschner Wires in Fixation of the Chevron Osteotomy for Treatment of Hallux Valgus Deformity: A Prospective Randomized Study with 2-Year Follow-Up. J. Orthop. Sports Med..

[115] Ciobanu I., Stanculescu (Badea) D.I., Iliescu A., Popescu A.M., Seiciu P.L., Mikolajczyk T., Moldovan F., Berteanu M. (2018). The Usability Pilot Study of a Mechatronic System for Gait Rehabilitation. Procedia Manuf..

